# Nomogram-Based Mortality Prediction in Acute Pulmonary Embolism Using Inflammatory Biomarkers and the Simplified Pulmonary Embolism Severity Index

**DOI:** 10.3390/jcm15124531

**Published:** 2026-06-11

**Authors:** Hacı Mehmet Çalışkan, Ömer Jaradat, Burak Şahin, Bilgehan Mutlu, Sedat Koçak, Sinem Deniz, Anılcan Kılıç, Alperen Yıldız, Veli Ağgül

**Affiliations:** 1 Faculty of Medicine, Department of Emergency Medicine, Kırşehir Ahi Evran University, 40100 Kırşehir, Türkiye; sinemozbingul@gmail.com (S.D.); anilcankilic@gmail.com (A.K.); dralperenyildiz@gmail.com (A.Y.); drveliaggul@gmail.com (V.A.); 2 Faculty of Medicine, Department of Emergency Medicine, Necmettin Erbakan University, 42080 Konya, Türkiye; dromerjaradat@gmail.com (Ö.J.); drskocak06@gmail.com (S.K.); 3 Faculty of Medicine, Department of Emergency Medicine, Ufuk University, 06530 Ankara, Türkiye; drburaksahin40@gmail.com; 4 Department of Emergency Medicine, Kırşehir Training and Research Hospital, 40100 Kırşehir, Türkiye; drbilgehanmtlu@gmail.com

**Keywords:** pulmonary embolism, inflammatory biomarkers, RDW-to-albumin ratio, platelet-to-lymphocyte ratio, nomogram, mortality prediction, sPESI, risk stratification, thrombo-inflammation pulmonary embolism, inflammatory biomarkers, RDW to albumin ratio, PLT/D-dimer ratio, nomogram, mortality prediction, sPESI, risk stratification, thrombo-inflammation

## Abstract

**Background/Objectives:** Pulmonary embolism (PE) remains a major cause of mortality, requiring rapid risk stratification. Widely used clinical tools such as the simplified Pulmonary Embolism Severity Index (sPESI) may not fully capture the disease’s inflammatory burden. This study aimed to evaluate the prognostic value of multiple inflammatory indices and to develop a clinically applicable nomogram integrating these indices with sPESI for mortality prediction in acute PE. **Methods:** This multicenter retrospective cohort study included 338 patients with acute PE. Ten inflammatory indices were calculated from admission laboratory data. The primary outcome was 12-month all-cause mortality; secondary outcomes were 30-day and 90-day mortality. Receiver operating characteristic analysis, multivariable Cox regression, and person-time analysis were performed. A composite inflammatory risk score (0–10) was developed, and a nomogram combining this score with sPESI was constructed. Internal validation used 1000 bootstrap resamples. **Results:** Overall mortality was 44.1%, with 41% of deaths occurring in the first 12 months. The red cell distribution width-to-albumin ratio (RAR) showed the highest discriminative performance (AUC = 0.755, 95% CI: 0.704–0.806). Each 1-point increase in the inflammatory risk score was independently associated with increased 30-day mortality (HR: 1.21, 95% CI: 1.10–1.34) and 90-day mortality (HR: 1.25, 95% CI: 1.15–1.36). The nomogram improved risk classification, particularly in patients with intermediate sPESI scores (1–2). The combined model achieved an AUC of 0.806 (95% CI: 0.761–0.851), with good calibration (Hosmer–Lemeshow *p* = 0.342). Platelet-to-lymphocyte ratio (PLR) did not show significant prognostic value. **Conclusions:** RAR is a strong, independent predictor of mortality in acute PE, providing incremental prognostic value beyond sPESI. The integrated nomogram enables more precise risk stratification and offers a practical, low-cost tool for bedside use.

## 1. Introduction

Acute pulmonary embolism (PE) is the most severe and potentially fatal clinical form of venous thromboembolism (VTE) and remains a major public health problem worldwide [1]. PE is the third most common acute cardiovascular syndrome after acute myocardial infarction and stroke, with an annual incidence ranging from approximately 39 to 115 cases per 100,000 population in the general population, and incidence rates increase markedly with age [1,2]. It is estimated that approximately 10–30% of VTE cases result in mortality within the first 30 days, although it is emphasized that a significant proportion of these deaths are preventable [3]. Appropriate anticoagulant therapy and hemodynamic stabilization can dramatically improve survival rates once PE is diagnosed [1,2]. However, its non-specific symptoms often complicate early diagnosis, underscoring the vital importance of a high clinical suspicion [1,2]. In this respect, PE is not merely a common pathology but a critical clinical condition requiring early diagnosis, meticulous risk stratification, and timely intervention.

Currently, the Pulmonary Embolism Severity Index (PESI) and its simplified version, sPESI, are widely used for risk stratification [4]. However, these clinical scores are largely based on demographic and clinical variables and may not fully reflect the biological processes of the disease. Recent studies have shown that acute PE is associated not only with mechanical obstruction of the pulmonary arteries but also with a pronounced thrombo-inflammatory process [5]. The inflammatory response that develops during thrombus formation contributes to clinical deterioration by increasing right ventricular load through mechanisms such as endothelial dysfunction, cytokine release, leukocyte activation, and platelet aggregation [6].

In line with these pathophysiological mechanisms, inflammatory indices derived from routine complete blood count (CBC) parameters are receiving increasing attention for prognostic assessment of PE. Markers such as the neutrophil-to-lymphocyte ratio (NLR), platelet-to-lymphocyte ratio (PLR) [7], systemic immune-inflammation index (SII), and systemic inflammation response index (SIRI) have been shown to reflect systemic inflammatory burden and are associated with mortality and disease severity [8]. Furthermore, composite biomarkers that combine inflammation with nutritional status or coagulation parameters, such as the red cell distribution width (RDW)-to-albumin ratio (RAR), monocyte-to-albumin ratio (MAR), C-reactive protein-to-albumin ratio (CAR), and platelet-to-D-dimer ratio, have also been reported to have prognostic value [9,10,11,12]. Although several studies have investigated individual inflammatory biomarkers in PE, evidence regarding the combined evaluation of multiple inflammatory indices and their integration with clinical risk scores remains limited.

Therefore, this study aimed to evaluate the prognostic value of various inflammatory indices for mortality in patients with acute PE, compare their performance with the sPESI score, and develop an integrated risk prediction model (nomogram) combining clinical and biochemical parameters. These biomarkers, which can be easily obtained from routine laboratory tests, may offer practical, low-cost prognostic tools that could improve early risk stratification in PE.

## 2. Materials and Methods

### 2.1. Study Design and Setting

This study was designed as a multicenter, retrospective cohort study and was conducted in the Emergency Departments (ED) of three tertiary care centers in Turkey: Necmettin Erbakan University Hospital, Kırşehir Education and Research Hospital, and Ufuk University Rıdvan Ege Hospital. The study protocol was reviewed and approved by Kırşehir Ahi Evran University Medical Faculty Ethics Committee (Approval No: 2025-14/178, Date: 9 September 2025). The study was conducted in accordance with the ethical principles of the Declaration of Helsinki. Reporting of the findings adheres to the STROBE statement (Table S1), and items pertinent to model performance were handled according to TRIPOD guidelines where applicable (Table S2). Patients diagnosed with PE in the ED between 1 September 2018 and 1 September 2024 were included. Survival data and mortality status were retrospectively followed up until 1 September 2025.

### 2.2. Study Population

Potential participants were screened based on predefined eligibility criteria. The inclusion criteria were: (1) age 18 years or older; and (2) confirmed diagnosis of acute PE via computed tomography pulmonary angiography. To ensure that the inflammatory indices specifically reflected the PE-related inflammatory state rather than other confounding conditions, the following exclusion criteria were applied: (1) presence of an active infection at the time of diagnosis; (2) known COVID-19 infection; (3) hematologic malignancy; (4) incomplete clinical or laboratory data; and (5) unconfirmed diagnosis of PE.

### 2.3. Data Collection

Patient data were retrospectively collected from hospitals’ information management systems and electronic patient records between 1 October 2025 and 1 February 2026. All patient identifiers were removed before analysis, and the authors had no access to identifiable information during data collection and analysis. The following variables were recorded: demographic data (age, sex), vital signs (systolic/diastolic blood pressure, heart rate, SpO_2_), and comorbidities (hypertension, diabetes mellitus, chronic obstructive pulmonary disease, chronic cardiovascular disease, malignancy, etc.). History of surgery within the last month and presence of deep vein thrombosis (DVT) were also documented. In addition, the 12-month mortality status for each patient was obtained from hospital records. All blood samples used for inflammatory indices were obtained within the first 4 h of ED presentation, representing the earliest available time point after symptom onset. Furthermore, data regarding all medications used and additional comorbidities were retrieved from the electronic health records. Patients whose medical records were incomplete were excluded from the study.

### 2.4. Outcomes

The prespecified primary outcome was 12-month all-cause mortality. Secondary outcomes included 30-day and 90-day all-cause mortality. Mortality status was ascertained from hospital records and regional health information systems.

### 2.5. Blood Sampling and Laboratory Analysis

Peripheral venous blood samples were systematically collected from all patients within 4 h of ED admission, prior to the initiation of definitive anticoagulant therapy. The following laboratory parameters were measured: RDW, serum albumin, D-dimer, troponin, and serum creatinine. Complete blood counts (CBC) were collected in ethylenediaminetetraacetic acid (EDTA) tubes and analyzed using automated hematology analyzers (Mindray BC-6800; Mindray Bio-Medical Electronics Co., Ltd., Shenzhen, China). RDW was measured as RDW-CV (%). Biochemical parameters, including serum albumin and C-reactive protein (CRP), were collected in gel-separator tubes, centrifuged at 3000 rpm for 10 min at room temperature, and processed using core laboratory chemical analyzers (Abbott Alinity c; Abbott Laboratories, Abbott Park, IL, USA). Across all three study centers, identical analytical platforms were used, and all assays were performed in strict accordance with the manufacturers’ operating instructions. Daily internal and external quality control procedures were followed at each participating center. Calculated inflammatory indices were derived using baseline absolute values obtained from these admission samples.

### 2.6. Measurement of Inflammatory Biomarkers and Indices

For all patients with a confirmed diagnosis of PE, inflammatory biomarkers and derived inflammatory indices were evaluated using laboratory parameters obtained at ED admission, including CBC and biochemical markers. The derived inflammatory indices were calculated as follows: NLR as neutrophil count (×10^3^/µL)/lymphocyte count (×10^3^/µL); PLR as platelet count (×10^3^/µL)/lymphocyte count (×10^3^/µL); MLR as monocyte count (×10^3^/µL)/lymphocyte count (×10^3^/µL); SII as platelet count × neutrophil count/lymphocyte count; SIRI as neutrophil count × monocyte count/lymphocyte count; CAR as CRP (mg/L)/serum albumin (g/dL); MAR as monocyte count (×10^3^/µL)/serum albumin (g/dL); RAR as RDW (%)/serum albumin (g/dL); platelet-to-D-dimer ratio as platelet count (×10^3^/µL)/D-dimer (µg/L); and mean platelet volume-to-D-dimer ratio (MPV/D-dimer) as MPV (fL)/D-dimer (µg/L).

### 2.7. sPESI

sPESI was calculated for each patient to assess the risk of 30-day mortality, as described by Jiménez et al. [4]. The score was determined based on the following six clinical variables: age > 80 years, presence of active cancer, chronic cardiopulmonary disease, heart rate ≥ 110 beats/min, systolic blood pressure < 100 mmHg, and SpO_2_ < 90%. Following the validated scoring system, one point was assigned for the presence of each variable. Patients were subsequently categorized into two risk groups: Low risk (sPESI = 0) and high risk (sPESI ≥ 1).

### 2.8. Statistical Analysis

Statistical analyses were performed using JAMOVI software (Version 2.6.44, The jamovi project, Sydney, Australia). Normality of continuous variables was assessed with the Shapiro–Wilk test. Variables that did not follow a normal distribution or had a limited number of categories were presented as median (interquartile range, IQR), while normally distributed variables were expressed as mean ± standard deviation (SD). Categorical variables were summarized as counts (*n*) and percentages (%). For group comparisons, the Chi-square (χ^2^) test or Fisher’s exact test (when appropriate) was used for categorical variables; the independent samples *t*-test for normally distributed continuous variables; and the Mann–Whitney U test for non-normally distributed continuous variables. The ability of inflammatory markers and risk indices to predict mortality was evaluated using ROC analysis. Performance was reported as Area Under the Curve (AUC) with 95% confidence intervals (CI). The Youden J index was used to determine the optimal cut-off values. Independent prognostic factors associated with mortality were identified using Cox proportional hazards regression. Multicollinearity among predictors was assessed before multivariable modeling. A *p*-value < 0.05 was considered statistically significant.

### 2.9. Nomogram Development and Internal Validation

A wide range of inflammatory indices was evaluated to avoid preselection bias and to identify the most discriminative parameters for the composite risk score. ROC analyses were initially performed to evaluate the discriminative performance of individual inflammatory biomarkers and indices for mortality prediction. Variables demonstrating significant prognostic performance were entered into multivariable Cox proportional hazards regression and binomial logistic regression models. Based on these evaluations, a composite inflammatory risk score was generated and integrated with the sPESI categories to construct a predictive nomogram for 12-month all-cause mortality.

In compliance with the TRIPOD guidelines for clinical prediction models, internal validation of the developed nomogram was performed using bootstrap resampling with 1000 repetitions. Model discrimination was quantified using Harrell’s Concordance Index (C-index), and an optimism-corrected C-index was calculated to adjust for potential overfitting. Calibration was evaluated and visualized using a bootstrap-corrected calibration plot with 95% confidence intervals (CI). Multicollinearity among predictors was assessed using the variance inflation factor (VIF), with a threshold of VIF < 5 indicating no significant collinearity. The proportional hazards assumption of the Cox regression model was tested using Schoenfeld residuals. Additionally, a binomial logistic regression model was fitted to evaluate the combined discriminative ability of the inflammatory risk score and sPESI for 12-month mortality, with results reported as area under the receiver operating characteristic curve (AUC), sensitivity, specificity, and accuracy. All statistical tests were two-sided, and a *p*-value < 0.05 was considered statistically significant.

## 3. Results

### 3.1. Patient Characteristics

**Figure 1:**
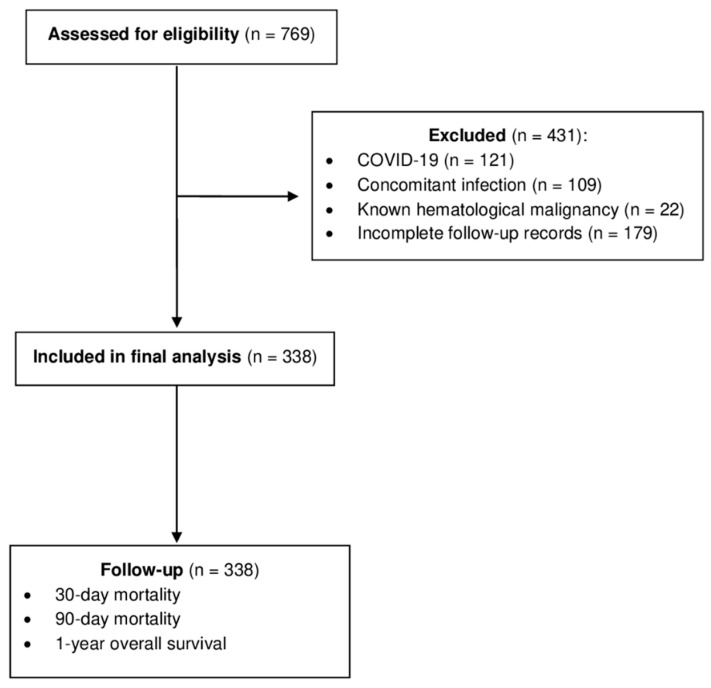
Participant flowchart.

Based on electronic medical records from participating centers, a total of 769 patients were initially identified with a diagnosis of PE during the study period. However, 431 patients were excluded from the study: 121 patients due to COVID-19, 22 due to hematologic malignancy, 109 due to concomitant infection, and 179 due to incomplete 12-month long-term survival follow-up records or inability to link data with the national electronic death registry, ensuring that missing-data bias was avoided and data integrity was maintained across all endpoints. Ultimately, 338 patients with complete data and follow-up records were included in the final analysis. The detailed screening process and the specific number of patients excluded at each stage are illustrated in the Participant Flowchart (Figure 1).

The mean age of the 338 patients included in the study was 66.7 ± 15.7 years. During the follow-up period, 44.1% of the patients died. Baseline sociodemographic and clinical characteristics were compared according to mortality status, and summarized in Table 1. The mean age of the non-survivor group (73.5 ± 12.3 years) was significantly higher than that of the survivors (61.4 ± 15.9 years). The prevalence of hypertension, diabetes mellitus, COPD, cardiovascular disease, and cancer was significantly higher in the non-survivor group. The proportion of patients with an sPESI score of 0 was markedly lower in the non-survivor group (7.4%) compared with the survivor group (30.2%), whereas the proportion with an sPESI score ≥ 2 was higher (29.5% vs. 6.9%). There were no statistically significant differences between the two groups regarding sex, history of DVT, history of surgery, echocardiographic findings, or receipt of thrombolytic therapy (Table 1).

When categorized according to clinical severity guidelines, 29.3% (*n* = 99) of the total cohort presented with massive PE, 46.2% (*n* = 156) with sub-massive PE, and 24.6% (*n* = 83) with low-risk PE. Clinical severity was fundamentally associated with long-term prognosis (*p* < 0.001). Notably, non-survivors exhibited a significantly higher prevalence of massive PE at presentation compared to survivors (44.3% vs. 17.5%), whereas low-risk PE cases were predominantly concentrated within the survivor group (39.7% vs. 5.4%). Regarding acute management strategies, the vast majority of patients received Low-Molecular-Weight Heparin (89.9%, *n* = 304) as their initial anticoagulation regimen, while 10.1% (*n* = 34) were treated with Unfractionated Heparin, demonstrating a homogeneous distribution between the survival groups (*p* = 0.146). Advanced reperfusion strategies were deployed based on clinical indications; systemic thrombolytic therapy was administered to 22.2% (*n* = 75) of the overall cohort, with a significantly higher utilization rate documented in the non-survivor group (32.9% vs. 13.8%, *p* < 0.001). Mechanical thrombectomy was performed in 7.4% (*n* = 25) of the patients, showing no statistical difference across the survival groups (*p* = 0.096). For long-term maintenance therapy, oral anticoagulation was prescribed to 70.4% (*n* = 238) of the total cohort. The use of maintenance oral anticoagulants was strongly tied to favorable survival outcomes, with survivors exhibiting a significantly higher rate of oral anticoagulant utilization compared to non-survivors (81.5% vs. 56.4%, *p* < 0.001) (Table 1).

During the follow-up period, many inflammatory parameters differed significantly between patients who died and those who survived. In the mortality group, NLR, MLR, MAR, RAR, SII, SIRI, and CAR were significantly higher. In contrast, the PLT/D-dimer and MPV/D-dimer ratios were significantly lower in patients who died. No significant difference in PLR was observed between the groups. When clinical risk scores were evaluated, the sPESI score was significantly higher in patients who died (Table 2).

### 3.2. Performance of Inflammatory Biomarkers and Indices

**Figure 2:**
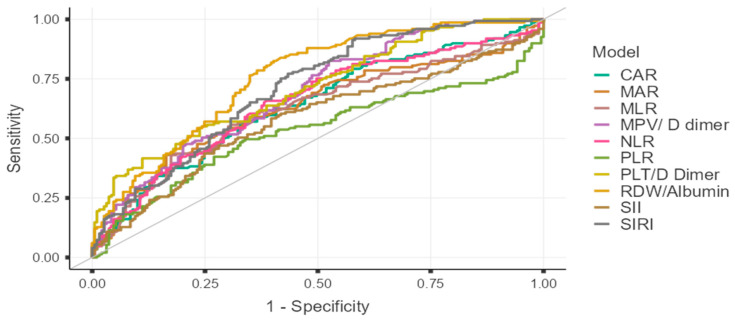
Receiver operating characteristic curves of inflammatory biomarkers and indices for prediction of mortality in acute pulmonary embolism.

The discriminative ability of inflammatory markers and risk indices to predict mortality was evaluated using ROC analysis. The results are presented as AUC values, optimal cut-off points, sensitivity, and specificity. The RDW/Albumin ratio had the highest AUC among all parameters, demonstrating the strongest discriminative performance for predicting mortality (AUC = 0.755, 95% CI: 0.704–0.806, *p* < 0.001). This value indicates an “acceptable” level of discrimination. The optimal cut-off point was determined as ≥3.80, and using this threshold, mortality could be predicted with 82.55% sensitivity and 59.79% specificity. The PLT/D-Dimer ratio stands out with the second highest AUC value (AUC = 0.710, 95% CI: 0.655–0.765, *p* < 0.001). This ratio also has an “acceptable” level of discriminative power. The optimal cut-off point is ≤39.31, yielding 55.03% sensitivity and 75.66% specificity. The MPV/D-Dimer ratio similarly showed good performance (AUC = 0.694, 95% CI: 0.638–0.749, *p* < 0.001). The optimal cut-off point was determined as ≤3.46, and with this value, mortality could be predicted with 82.55% sensitivity and 47.62% specificity. SIRI showed a moderate but significant discriminative power (AUC = 0.709, 95% CI: 0.655–0.764, *p* < 0.001). The optimal cut-off point is ≥1.58, providing 77.18% sensitivity and 56.61% specificity. Other inflammatory ratios, such as NLR, MLR, MAR, and CAR, had AUC values ranging from 0.623 to 0.650, which were statistically significant (*p* < 0.001) but had weak-to-moderate discriminative ability. SII showed weak but significant performance (AUC = 0.579, *p* = 0.014). For PLR (Platelet-Lymphocyte Ratio), the AUC value was calculated as 0.529, and this result was not statistically significant (*p* = 0.383). PLR was found to be insufficient for discriminating mortality (Table 3, Figure 2).

### 3.3. Correlations Between Inflammatory Biomarkers and sPESI

The correlation between inflammatory indices and the sPESI score is presented in Table 3. NLR (r = 0.198, *p* < 0.001), RDW/albumin ratio (r = 0.294, *p* < 0.001), SIRI (r = 0.217, *p* < 0.05), and CAR (r = 0.126, *p* < 0.05) showed statistically significant positive correlations with the sPESI score. The PLT/D-dimer (r = −0.292, *p* < 0.001) and MPV/D-dimer (r = −0.210, *p* < 0.001) ratios demonstrated significant negative correlations with sPESI (Table 4).

### 3.4. Person-Time Mortality Analysis

Mortality incidence during follow-up was evaluated using person-time analysis. Over a total follow-up of 196,157 months (approximately 16,346 patient-years), 149 deaths were observed, yielding an overall incidence rate of 0.08 per 100 person-months (95% CI: 0.06–0.09). When the distribution of mortality incidence over time intervals was examined, the risk decreased markedly over time. The highest incidence rate was observed in the first 12 months after diagnosis (2.31 per 100 person-months; 95% CI: 1.77–2.97), with approximately 41% of all deaths (61/149) occurring during this period. From the second year onward, the incidence rate decreased substantially: 0.49 (95% CI: 0.33–0.70) between 12–36 months, and 0.14 (95% CI: 0.06–0.28) between 36–60 months. After 60 months, the incidence rate fell to its lowest level of 0.03 per 100 person-months (95% CI: 0.02–0.04). These findings indicate that the highest risk of mortality in patients with PE occurs during the first 12 months, with risk gradually decreasing in subsequent years. Close follow-up of patients, particularly during the first year, is critical for reducing mortality (Table 5).

### 3.5. Predictors of 30-Day and 90-Day Mortality

**Figure 3:**
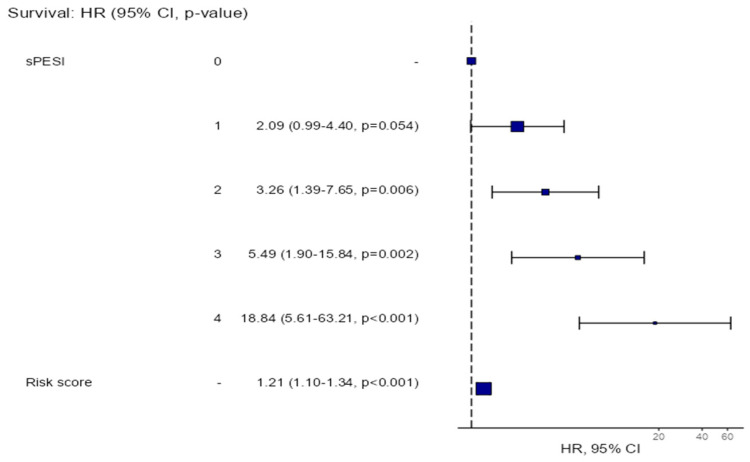
Forest plot of multivariable Cox regression analysis for 30-day mortality in patients with acute pulmonary embolism. The square boxes represent the point estimates of the Hazard Ratios (HR), the horizontal lines represent the corresponding 95% confidence intervals (CI), and the vertical dashed line indicates the line of null effect (HR = 1.0).

Factors predicting 30-day and 90-day mortality in patients with PE are presented in Table 6. In multivariable Cox regression analysis, both categorical levels of the sPESI score and the continuous inflammatory risk score were independent predictors of mortality at both time points. For 30-day mortality, compared with the reference group (sPESI 0), patients with sPESI 1 had a 2.09-fold increased risk (95% CI: 0.99–4.40, *p* = 0.054), which was not statistically significant. Patients with sPESI 2 had a 3.26-fold increased risk (95% CI: 1.39–7.65, *p* = 0.006); sPESI 3: 5.49-fold (95% CI: 1.90–15.84, *p* = 0.002); and sPESI 4: 18.84-fold (95% CI: 5.61–63.21, *p* < 0.001). Each one-unit increase in the inflammatory risk score increased the 30-day mortality risk by 21% (HR: 1.21, 95% CI: 1.10–1.34, *p* < 0.001) (Figure 3).

**Figure 4:**
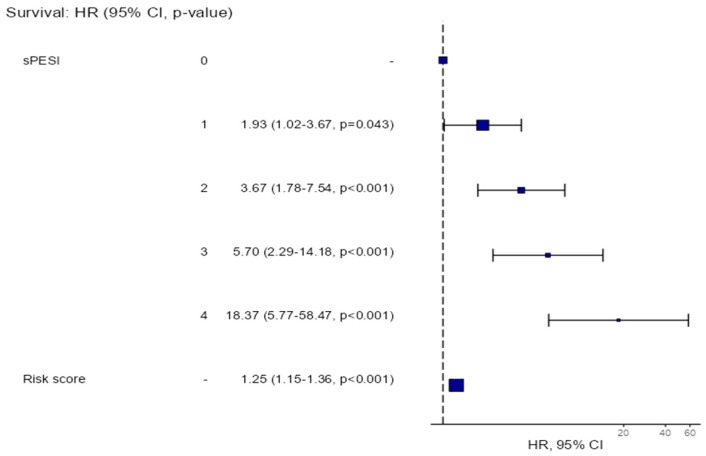
Forest plot of multivariable Cox regression analysis for 90-day mortality in patients with acute pulmonary embolism. The square boxes represent the point estimates of the Hazard Ratios (HR), the horizontal lines represent the corresponding 95% confidence intervals (CI), and the vertical dashed line indicates the line of null effect (HR = 1.0).

For 90-day mortality, patients with sPESI 1 had a 1.93-fold increased risk (95% CI: 1.02–3.67, *p* = 0.043); sPESI 2: 3.67-fold (95% CI: 1.78–7.54, *p* < 0.001); sPESI 3: 5.70-fold (95% CI: 2.29–14.18, *p* < 0.001); and sPESI 4: 18.37-fold (95% CI: 5.77–58.47, *p* < 0.001). Each one-unit increase in the inflammatory risk score increased the 90-day mortality risk by 25% (HR: 1.25, 95% CI: 1.15–1.36, *p* < 0.001). These findings demonstrate that both sPESI and the inflammatory risk score are strong markers for stratifying short- and intermediate-term mortality in patients with PE (Table 6, Figure 4).

### 3.6. Development of the Predictive Nomogram

**Figure 5:**
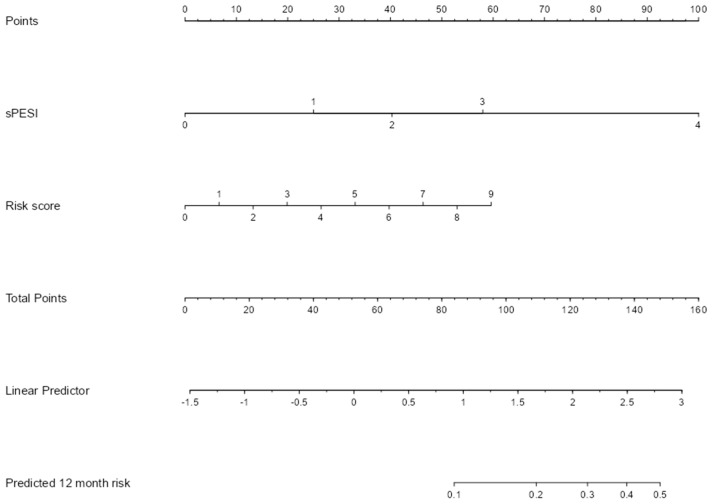
Nomogram integrating the composite inflammatory risk score and sPESI for prediction of 12-month mortality in patients with acute pulmonary embolism.

To develop a predictive nomogram, we performed univariable and multivariable Cox regression analyses using the inflammatory indices that demonstrated significant discriminatory power in ROC analysis (AUC > 0.6 and *p* < 0.05) (Table 3, Figure 2). Variables that remained independently associated with mortality in the multivariable model were selected as predictors. Based on these cut-off values, a categorical score was assigned to each patient: 1 point was given if an inflammatory index value was above the determined cut-off (or below the cut-off for the PLT/D-dimer and MPV/D-dimer indices), and 0 points otherwise. Subsequently, the total risk score was calculated by summing the points obtained from the ten inflammatory indices. This total risk score theoretically ranges from 0 to 10, with higher scores indicating an increased inflammatory burden and consequently a higher risk of mortality. Based on the multivariable Cox regression analysis, a nomogram was developed to predict 12-month mortality in patients with PE (Figure 5). In the nomogram, the contribution of each variable is expressed as points. For the sPESI score, point assignments were: sPESI 0 = 0 points, sPESI 1 = 25 points, sPESI 2 = 40 points, sPESI 3 = 58 points, and sPESI 4 = 100 points. For the inflammatory risk score, although its theoretical range is 0–10, no patient in our cohort had a score of 10; therefore, the nomogram is based on the observed range of 0–9. Point assignments for the observed scores were: 0→0, 1→7, 2→13, 3→20, 4→26, 5→33, 6→40, 7→46, 8→53, and 9→60 points. The sum of points from both variables gives the patient’s total point score, and the corresponding 12-month mortality risk is read from the nomogram. Accordingly, a patient with a total point score of 84 has a 12-month mortality risk of 10%; a score of 109 corresponds to 20% risk; 125 points → 30%; 138 points → 40%; and 148 points → 50% risk. Notably, an sPESI score of 4 alone contributes 100 points, nearly the maximum, indicating that these patients are at very high risk. The inflammatory risk score allows more precise risk stratification, especially in patients with intermediate sPESI scores. This nomogram is a practical and reliable tool for estimating individual 12-month mortality risk in patients with PE.

### 3.7. Internal Validation and Model Diagnostics

**Figure 6:**
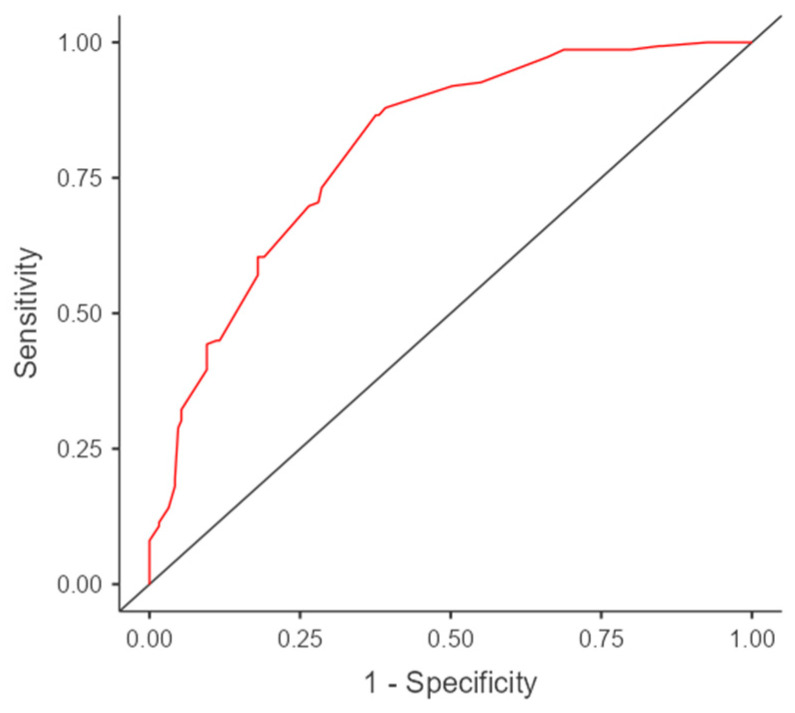
Receiver operating characteristic (ROC) curve of the binomial logistic regression model combining the total inflammatory risk score and sPESI categories for predicting 12-month mortality. The solid red line represents the combined predictive model, and the dashed diagonal line represents the reference line of random chance.

The multivariable binomial logistic regression model constructed to evaluate 12-month mortality demonstrated an excellent overall fit (Deviance = 358, AIC = 370, BIC = 393, McFadden’s R^2^ = 0.228) and was statistically highly significant (Omnibus likelihood ratio test χ^2^ = 106, df = 5, *p* < 0.001). Table 7 presents the detailed coefficients and model fit statistics. The total inflammatory risk score emerged as a powerful independent predictor; each 1-unit increase expanded the odds of death by 54.4% (OR: 1.544, 95% CI: 1.359–1.755, *p* < 0.001). Compared to patients with sPESI = 0, those with sPESI = 1 (OR: 3.426, 95% CI: 1.613–7.278, *p* = 0.001), sPESI = 2 (OR: 12.041, 95% CI: 4.288–33.813, *p* < 0.001), and sPESI = 3 (OR: 14.443, 95% CI: 3.177–65.650, *p* < 0.001) exhibited exponentially higher mortality risks. Multicollinearity analysis confirmed the complete independence of the predictors (VIF = 1.00 for all predictors), ensuring mathematical stability. The proportional hazards assumption of the Cox regression model was satisfied globally (Schoenfeld test: χ^2^ = 9.04, df = 5, *p* = 0.107); although the risk score alone showed a borderline violation (*p* = 0.010), the global non-significant result supports the overall adequacy of the model. At a clinical cut-off of 0.5, the integrated model yielded an outstanding AUC of 0.806 (95% CI: 0.761–0.851), with an accuracy of 71.9%, sensitivity of 60.4%, and specificity of 81.0% (Figure 6).

**Figure 7:**
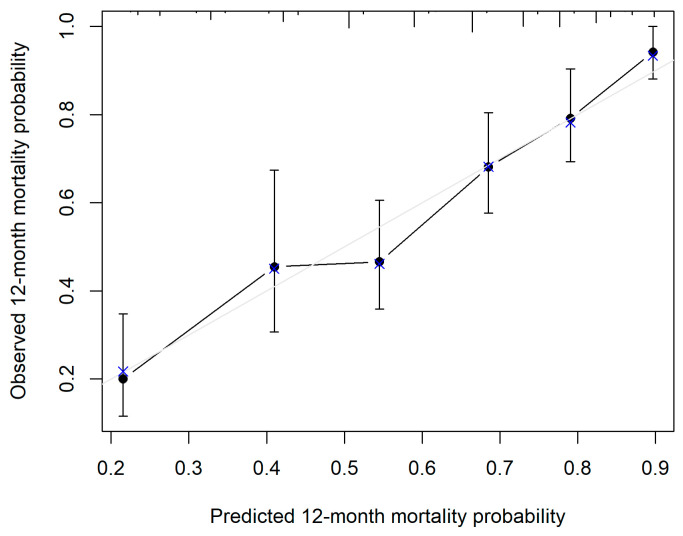
Bootstrap-corrected calibration plot for the nomogram predicting 12-month mortality. The calibration curve was generated using 1000 bootstrap resamples. The solid black line represents the bias-corrected predicted probabilities, and the diagonal line indicates perfect calibration (predicted = observed). The vertical error bars at each point represent the 95% confidence intervals. The non-significant Hosmer–Lemeshow test (*p* = 0.342) supports good agreement between predicted and observed 12-month mortality risks across the entire risk spectrum.

For internal validation of the nomogram, 1000 bootstrap resamples were used. The nomogram demonstrated an apparent Harrell’s C-index of 0.730; after optimism correction, the C-index was 0.725, indicating minimal overfitting and acceptable discriminative performance. The bootstrap-corrected calibration plot showed good agreement between predicted and observed 12-month mortality probabilities, supported by a Hosmer–Lemeshow test (*p* = 0.342) (Figure 7).

## 4. Discussion

In this multicenter retrospective cohort study, RAR demonstrated the highest discriminative performance for predicting mortality among all evaluated inflammatory biomarkers and indices, with an AUC of 0.755. The study revealed that mortality risk in patients with PE is highest during the first 12 months, with approximately 41% of all deaths occurring in this critical period. Our cohort consisted of tertiary care centres with a high comorbidity burden, and the follow-up extended to 12 months, which explains the higher mortality compared to studies focusing only on in-hospital or 30-day outcomes. Furthermore, each one-unit increase in the “Total Inflammatory Risk Score” (composed of ten different inflammatory indices) was found to increase the risk of 30-day mortality by 21% independently and the risk of 90-day mortality by 25%. The developed nomogram model significantly improved the predictive power of sPESI alone, particularly by refining risk classification in patients with intermediate sPESI scores (1–2). Internal validation using 1000 bootstrap resamples confirmed the nomogram’s robustness, with an optimism-corrected Harrell’s C-index of 0.725 and good calibration. On the other hand, the fact that PLR, which has been extensively studied in the literature, did not show statistically significant performance in discriminating mortality in this cohort is another notable negative finding.

Consistent with our results, two studies in the literature have reported that RAR is strongly associated with both in-hospital and long-term mortality among PE patients [13,14]. However, there are marked differences in reported cut-off values. While the optimal cut-off in our study was ≥3.80, Eraslan et al. reported 5.294, and Başyiğit et al. reported a higher value of 13.6 [10,14]. The main reason for this discrepancy may be that some studies use RDW-SD (fL) while others use RDW-CV (%) for RDW measurement. This highlights the importance of standardizing the unit used in RAR calculation for clinical interpretation. The strong prognostic performance of RAR can be explained by its ability to integrate the pathophysiology of PE: RDW reflects oxidative stress and erythrocyte heterogeneity, while albumin is an indicator of inflammation and nutritional status.

The second notable finding of our study is that the PLT/D-dimer ratio emerged as the second strongest parameter (AUC = 0.710), and our composite risk score significantly enhanced the predictive power of sPESI. In line with our study, Li et al. [11] demonstrated that the D-dimer-to-platelet ratio performed well (AUC = 0.721) in predicting in-hospital adverse events and significantly improved model accuracy when added to the sPESI score (*p* < 0.001). Li et al. [11] attributed an elevated ratio to increased thrombus burden and right ventricular dysfunction. Correspondingly, our finding of elevated D-dimer and decreased platelets in the mortality group (*p* < 0.001) highlights a critical clinical shift. It confirms the intense balance between coagulation activation and platelet consumption in PE pathophysiology, playing a decisive role in patient survival. Huang et al. [15] showed that the combination of MPV and D-dimer outperformed D-dimer alone in the diagnosis of PE. This synergistic approach, proposed by Huang et al. for diagnostic purposes, gains a prognostic dimension in our study through the success of PLT/D-dimer (AUC: 0.710) and MPV/D-dimer (AUC: 0.694) ratios in predicting mortality.

Our study also yielded significant results for NLR, one of the most frequently investigated inflammatory markers (*p* < 0.001). This finding is consistent with the meta-analysis of 15 studies by Tang and Hu [7] and the study by Babes et al. [16]. However, the discriminative ability of NLR (AUC = 0.650) was lower than that of RAR. This suggests that indices based on a single parameter, reflecting only cellular inflammation, may have more limited prognostic value than composite indicators that evaluate both inflammation and nutritional status.

On the other hand, the lack of a significant association between PLR and mortality in our study (*p* = 0.383) provides an important clarification to the conflicting results in the literature. Notably, the meta-analysis by Tang and Hu [7] showed that PLR has no independent role in predicting PE mortality (OR: 1.00). The consistency of our results with this meta-analysis suggests that PLR may not be a reliable prognostic marker on its own in the clinical management of PE.

Although SII, which has gained increasing attention in recent years, was significantly higher in the mortality group in our study (*p* = 0.013), its discriminative ability remained limited (AUC = 0.579). In contrast, Mermer et al. [17] and Suwadi et al. [18] reported higher AUC values for SII. A possible reason for this discrepancy is that SII has been specifically associated with right ventricular dysfunction and high lactate levels in those studies. The longer follow-up period and the use of person-time analysis in our study suggest that SII may be a marker reflecting predominantly acute inflammatory burden. In contrast, SIRI’s stronger performance (AUC = 0.709) suggests that the role of monocytes in the thrombo-inflammatory process may be more decisive for PE prognosis. We deliberately evaluated multiple indices to avoid preselection bias and to determine the optimal composite score. The final nomogram integrates the ten-item risk score, which outperformed individual markers in predicting 12-month mortality.

Another recent study emphasizing the importance of SIRI and SII in PE management, by Özdemir et al. [19], reported that these indices have significant performance in identifying high-risk PE patients. Particularly noteworthy is that the AUC value for NLR in predicting high-risk embolism in Özdemir et al.’s study (0.650) exactly matches the value we obtained for mortality prediction in our study (0.650). Özdemir et al. noted that the single use of SIRI and SII could not maintain independence in multivariate analyses. In our study, converting SIRI (AUC = 0.709) into a risk score by combining it with other inflammatory markers demonstrates how the limited prognostic power of these parameters alone can provide significant added value to the sPESI score within a composite model (21–25% increase in mortality risk).

Another important marker examined in our study, CAR, has been associated with PE prognosis in the literature. Özcan et al. [12] reported that CAR is an independent predictor of 6-month mortality in PE patients and correlates with the PESI score. While Özcan et al. reported a higher AUC (0.763) for CAR with a cut-off of 5.33, our study found a lower discriminative ability (AUC = 0.638) and a higher optimal cut-off (≥12.69). This supports the justification for the ‘biomarker combinations’ approach proposed by Özcan et al. to enhance the clinical applicability of CAR. Indeed, rather than evaluating CAR alone, we aimed to increase the predictive power of sPESI and provide a more dynamic prognostic model by incorporating CAR into our 10-component risk score.

The use of nomograms in PE management is becoming increasingly common in the literature. For example, Liuyi et al. [20] developed a diagnostic nomogram to predict PE risk in oncology patients, including parameters such as neutrophil count, systolic blood pressure, surgical status, and D-dimer. That study showed that neutrophil count is an important marker of increased risk of PE. This finding is conceptually parallel to our results demonstrating the role of inflammatory indices on mortality. However, while Liuyi et al.’s study focused on the risk of developing PE, our study contributes to a different stage of clinical management by focusing on predicting mortality in patients already diagnosed with PE.

Another recent study supporting the value of nomograms in PE management, by Liu et al. [21], showed that their model for predicting PE diagnosis in elderly patients performed significantly better (AUC = 0.763) than the Wells and revised Geneva scores. Furthermore, the association of hematological indices such as MCHC included in Liu et al.’s nomogram with PE risk conceptually parallels the strongest prognostic performance of RAR in our study, reinforcing the importance of changes in erythrocyte morphology in the thrombo-inflammatory process.

Zhou et al. [22], in 2026, conducted a nomogram study in PE patients hospitalized in the intensive care unit. They reported that a nomogram model incorporating inflammatory markers such as NLR, LMR, and RDW provided higher discriminative ability (AUC = 0.772) for predicting mortality than the PESI (AUC = 0.686) and sPESI (AUC = 0.624) scores. These results strongly support our findings that the inflammatory risk score provides additional prognostic value to sPESI. Moreover, Zhou et al.’s identification of RDW > 14.35 as an independent risk factor for mortality aligns with RAR having the highest AUC value in our study. These results indicate that RDW is a central biomarker reflecting chronic inflammation, oxidative stress, and erythrocyte deformability. Consistent with this, the binomial logistic regression model combining the risk score and sPESI achieved an AUC of 0.806, further supporting the excellent discriminative ability of our nomogram.

Another critical clinical nuance regarding risk assessment tools involves the oncological patient population. Active malignancy is a core component of the sPESI scoring architecture, automatically assigning at least 1 point to any patient with cancer. Consequently, cancer patients with asymptomatic or incidentally detected PE are automatically categorized as “high risk” by sPESI [4]. This automatic up-scoring drastically reduces the specificity of sPESI in cancer sub-populations, leading to potential over-triaging. Our integrated nomogram offers a significant clinical solution to this diagnostic dilemma. By incorporating objective systemic inflammatory biomarkers like the RAR, the nomogram evaluates the actual acute physiological and biological stress response triggered by the thromboembolic event rather than relying purely on static clinical criteria. Therefore, an oncological patient with an incidental, low-burden PE and minimal systemic inflammatory disruption will yield a lower composite risk score, allowing clinicians to make a more personalized, granular, and safe disposition decision.

Many studies in the literature emphasize that although the sPESI score is successful in identifying low-risk patients, it may be limited in distinguishing intermediate- and high-risk patients [23,24,25]. One of the most important contributions of our study is that the 10-component inflammatory risk score we developed can fill this gap. This demonstrates that inflammatory processes play a central role in the prognosis of PE. Furthermore, the developed nomogram allows more precise risk classification, especially in patients with intermediate sPESI scores. For example, while patients with high sPESI scores are already in the high-risk group, our model shows that patients with sPESI scores of 1–2 but a high inflammatory score may carry a similar mortality risk. This approach offers a practical and low-cost solution to the frequently expressed need for more accurate risk stratification in the literature.

### Limitations

This study has several limitations. The retrospective design and single-country setting limit generalizability and causal inference. A considerable number of patients were excluded due to missing data, which may affect representativeness. Although internal validation (1000 bootstrap resamples) yielded an optimism-corrected C-index of 0.725, external validation in an independent cohort is lacking. Variability in RDW units (SD vs. CV) may affect comparability of RAR cut-offs; we used RDW-CV (%). Unmeasured confounders (e.g., medications, symptom-to-blood-draw time) could not be fully controlled. Finally, our cohort included asymptomatic cancer patients with incidental PE, for whom sPESI automatically scores ≥ 1 due to the cancer criterion, potentially overestimating risk; this subgroup requires specific validation of our nomogram.

## 5. Conclusions

In conclusion, our study confirmed that RAR is a strong prognostic biomarker in the management of PE and revealed the limited clinical value of PLR. The Total Risk Score and nomogram we developed enhance the clinical performance of sPESI, providing physicians with a rapid, low-cost, and highly accurate risk prediction tool for bedside use. The use of such inflammatory models may help reduce PE mortality, particularly in settings where advanced imaging or echocardiography facilities are limited.

## Figures and Tables

**Table 1:** Baseline sociodemographic and clinical characteristics of patients with acute pulmonary embolism.

Variables		Mortality *n* (%) or Mean ± SD or Median (25 to 75 Percentile)		
		No 189 (55.9)	Yes 149 (44.1)	*p*
Age		61.4 ± 15.9)	73.5 ± 12.3	<0.001
Sex	Male	77 (40.7%)	58 (38.9)	0.821
	Female	112 (59.3)	91 (61.1)	
Hypertension (HT)	No	96 (50.8)	58 (38.9)	0.039
	Yes	93 (49.2)	91 (61.1)	
Diabetes mellitus (DM)	No	138 (73.0)	94 (63.1)	0.033
	Yes	51 (27.0)	55 (36.9)	
Chronic obstructive Pulmonary Disease (COPD)	No	159 (84.1)	109 (73.2)	0.019
	Yes	30 (15.9)	40 (26.8)	
Cardiovascular disease (CVD)	No	179 (94.7)	117 (78.5)	<0.001
	Yes	10 (5.3)	32 (21.5)	
Deep vein thrombosis (DVT)	No	145 (76.7)	126 (84.6)	0.097
	Yes	44 (23.3)	23 (15.4)	
Operation history	No	157 (83.1)	119 (79.9)	0.539
	Yes	32 (16.9)	30 (20.1)	
Cancer	No	170 (89.9)	106 (71.1)	<0.001
	Yes	19 (10.1)	43 (28.9)	
Clinical Severity Classification	Massive PE	33 (33.3)	66 (66.7)	<0.001
	Sub-massive PE	81 (51.9)	75 (48.1)	
	Low-risk PE	75 (90.4)	8 (9.6)	
Anticoagulation Regimen	LMWH	166 (54.6)	138 (45.4)	0.146
	UFH	23 (67.6)	11 (32.4)	
Oral Anticoagulation (NOAC/VKA)	No	35 (18.5)	65 (43.6)	<0.001
	Yes	154 (81.5)	84 (56.4)	
Thrombolytic treatment	No	163 (86.2)	128 (85.9)	1.000
	Yes	26 (13.8)	21 (14.1)	
Mechanical Throm-bectomy	No	179 (57.2)	134 (42.8)	0.096
	Yes	10 (40.0)	15 (60.0)	
sPESI	0	57 (30.2)	11 (7.4)	<0.001
	1	119 (63.0)	94 (63.1)	
	2	10 (5.3)	31 (20.8)	
	3	3 (1.6)	9 (6.0)	
	4	0 (0.0)	4 (2.7)	

Data are presented as mean ± SD, *n* (%), or median (interquartile range, IQR). *p*-values: Chi-square test for categorical variables; independent samples *t*-test for normally distributed continuous variables (age only); Mann–Whitney U test for non-normally distributed continuous variables. COPD = chronic obstructive pulmonary disease; LMWH = Low-Molecular-Weight Heparin; PE = Pulmonary Embolism; sPESI = simplified Pulmonary Embolism Severity Index; UFH = Unfractionated Heparin; NOAC = Novel Oral Anticoagulant; VKA = Vitamin K Antagonist; SD = standard deviation.

**Table 2:** Admission laboratory parameters and derived inflammatory indices stratified by 12-month mortality.

Variables	Mortality *n* (%) or Mean ± SD or Median (25 to 75 Percentile)		
	No 189 (55.9)	Yes 149 (44.1)	*p*
NLR	3.4 (2.3 to 6.2)	5.4 (3.4 to 10.1)	<0.001
PLR	125.6 (88.9 to 198.2)	156.5 (73.6 to 255.0)	0.359
MLR	0.4 (0.2 to 0.5)	0.5 (0.3 to 0.8)	<0.001
MAR	0.2 (0.1 to 0.2)	0.2 (0.2 to 0.3)	<0.001
CAR	6.2 (2.3 to 19.0)	15.0 (4.6 to 39.5)	<0.001
RDW/Albumin	3.6 (3.2 to 4.4)	4.6 (3.9 to 5.9)	<0.001
SII	801.4 (491.0 to 1521.1)	1185.9 (541.8 to 2095.8)	0.013
SIRI	6.2 (2.3 to 19.0)	15.0 (4.6 to 39.5)	<0.001
PLT/D-Dimer	74.9 (39.7 to 147.6)	36.1 (18.4 to 75.8)	<0.001
MPV/D-dimer	3.2 (1.9 to 5.9)	1.8 (1.1 to 3.0)	<0.001
sPESI	1.0 (0.0 to 1.0)	1.0 (1.0 to 2.0)	<0.001

Data are presented as mean ± SD, *n* (%), or median (interquartile range, IQR). *p*-values: Chi-square test for categorical variables; independent samples *t*-test for normally distributed continuous variables (age only); Mann–Whitney U test for non-normally distributed continuous variables. NLR = neutrophil-to-lymphocyte ratio; PLR = platelet-to-lymphocyte ratio; MLR = monocyte-to-lymphocyte ratio; MAR = monocyte-to-albumin ratio; RDW = red cell distribution width; SII = systemic immune-inflammation index; SIRI = systemic inflammation response index; CAR = C-reactive protein-to-albumin ratio; PLT = platelet; MPV = mean platelet volume; SD = standard deviation.

**Table 3:** Performance of inflammatory indices for mortality classification.

Parameter	AUC	Std. Error	95% Confidence Interval		*p*	Optimal Cut-Off	Sensitivity	Specificity
			Lower	Upper				
NLR	0.650	0.0304	0.591	0.710	<0.001	≥4.15	65.77	61.38
PLR	0.529	0.0333	0.464	0.594	0.383	≥164.12	49.66	66.14
MLR	0.623	0.0315	0.561	0.685	<0.001	≥0.587	42.95	82.54
MAR	0.633	0.0314	0.571	0.694	<0.001	≥0.25	42.95	83.60
RDW/Albumin	0.755	0.0260	0.704	0.806	<0.001	≥3.80	82.55	59.79
SII	0.579	0.0321	0.516	0.642	0.014	≥1345	46.98	71.96
SIRI	0.709	0.0277	0.655	0.764	<0.001	≥1.58	77.18	56.61
CAR	0.638	0.0305	0.578	0.698	<0.001	≥12.69	55.70	67.20
PLT/D-Dimer	0.710	0.0279	0.655	0.765	<0.001	≤39.31	55.03	75.66
MPV/D-dimer	0.694	0.0282	0.638	0.749	<0.001	≤3.46	82.55	47.62

AUC = area under the curve. Optimal cut-off values were determined using the Youden J index. NLR = neutrophil-to-lymphocyte ratio; PLR = platelet-to-lymphocyte ratio; MLR = monocyte-to-lymphocyte ratio; MAR = monocyte-to-albumin ratio; SII = systemic immune-inflammation index; SIRI = systemic inflammation response index; CAR = C-reactive protein-to-albumin ratio; PLT = platelet; MPV = mean platelet volume.

**Table 4:** Correlations between inflammatory indices and the sPESI score.

Parameter	sPESI
NLR	0.198 ***
PLR	0.070
MLR	0.098
MAR	−0.017
RDW/Albumin	0.294 ***
SII	0.069
SIRI	0.217 *
CAR	0.126 *
PLT/D-Dimer	−0.292 ***
MPV/D-dimer	−0.210 ***

*** *p* < 0.001, * *p* < 0.05. Correlations were calculated using Spearman’s rank correlation coefficient (non-parametric) because most indices were not normally distributed. NLR = neutrophil-to-lymphocyte ratio; PLR = platelet-to-lymphocyte ratio; MLR = monocyte-to-lymphocyte ratio; MAR = monocyte-to-albumin ratio; SII = systemic immune-inflammation index; SIRI = systemic inflammation response index; CAR = C-reactive protein-to-albumin ratio; PLT = platelet; MPV = mean platelet volume.

**Table 5:** Person-Time Analysis.

Time Interval	Events (*n*)	Person-Time	Incidence Rate *	95% CI	
				Lower	Upper
Overall (0–max)	149	196,157.000	0.080	0.060	0.090
0–12	61	2635.000	2.310	1.770	2.970
12–36	30	6138.000	0.490	0.330	0.700
36–60	8	5634.000	0.140	0.060	0.280
60–84	50	181,750.000	0.030	0.020	0.040

* Incidence rate = (number of events/person-time) × 100. Person-time is expressed in months. The overall incidence rate is calculated as 149/196,157 × 100 = 0.076, rounded to 0.08. CI = confidence interval.

**Table 6:** Prediction of 30-day and 90-day mortality according to sPESI and inflammatory risk score.

30-Day Mortality		All	HR (Univariable)	HR (Multivariable)
sPESI	0	68 (20.1)	-	-
	1	213 (63.0)	2.50 (1.19–5.25, *p* = 0.015)	2.09 (0.99–4.40, *p* = 0.054)
	2	41 (12.1)	4.69 (2.04–10.80, *p* < 0.001)	3.26 (1.39–7.65, *p* = 0.006)
	3	12 (3.6)	6.17 (2.14–17.80, *p* = 0.001)	5.49 (1.90–15.84, *p* = 0.002)
	4	4 (1.2)	26.62 (7.97–88.90, *p* < 0.001)	18.84 (5.61–63.21, *p* < 0.001)
Risk score	Mean (SD)	4.3 (2.2)	1.25 (1.14–1.37, *p* < 0.001)	1.21 (1.10–1.34, *p* < 0.001)
90-Day Mortality				
sPESI	0	68 (20.1)	-	-
	1	213 (63.0)	2.35 (1.25–4.44, *p* = 0.008)	1.93 (1.02–3.67, *p* = 0.043)
	2	41 (12.1)	5.43 (2.68–11.00, *p* < 0.001)	3.67 (1.78–7.54, *p* < 0.001)
	3	12 (3.6)	6.31 (2.54–15.69, *p* < 0.001)	5.70 (2.29–14.18, *p* < 0.001)
	4	4 (1.2)	26.52 (8.35–84.22, *p* < 0.001)	18.37 (5.77–58.47, *p* < 0.001)
Risk score	Mean (SD)	4.3 (2.2)	1.28 (1.19–1.39, *p* < 0.001)	1.25 (1.15–1.36, *p* < 0.001)

Data are *n* (%) for sPESI categories or mean ± SD for risk score; HR (95% CI) from Cox regression; multivariable model includes sPESI and risk score; reference: sPESI = 0; risk score range 0–10 (observed 0–9); statistical significance defined as *p* < 0.05. HR = Hazard Ratio; sPESI = Simplified Pulmonary Embolism Severity Index; SD = Standard Deviation.

**Table 7:** Multivariable Binomial Logistic Regression for 12-Month Mortality.

Predictor	Beta (Estimate)	SE	Z	*p*	Odds Ratio (95% CI)
Intercept	−3.429	0.4665	−7.349	<0.001	0.032 (0.013–0.081)
Total Inflammatory Risk Score	0.434	0.0653	6.650	<0.001	1.544 (1.359–1.755)
sPESI Categories (Ref: 0)					
sPESI 1	1.231	0.3844	3.203	0.001	3.426 (1.613–7.278)
sPESI 2	2.488	0.5268	4.723	<0.001	12.041 (4.288–33.813)
sPESI 3	2.670	0.7725	3.456	<0.001	14.443 (3.177–65.650)

AIC = 370, BIC = 393, McFadden’s R^2^ = 0.228. Omnibus Likelihood Ratio Test: χ^2^ = 106, df = 5, *p* < 0.001. Multicollinearity: VIF = 1.00 for all predictors. sPESI category 4 (*n* = 4, all died) was excluded from logistic regression due to perfect separation; in Cox regression, sPESI 4 remained significantly associated with mortality (HR: 18.84, 95% CI: 5.61–63.21, *p* < 0.001). SE = standard error; CI = confidence interval; sPESI = simplified Pulmonary Embolism Severity Index.

## Data Availability

The raw data supporting the conclusions of this article will be made available by the authors upon request.

## Supplementary Materials

The following supporting information can be downloaded at: https://www.mdpi.com/article/10.3390/jcm15124531/s1, Table S1: STROBE Statement—checklist of items that should be included in reports of observational studies; Table S2: TRIPOD Checklist: Prediction Model Development and Validation.
