# Supplementary material for: Nomogram-Based Mortality Prediction in Acute Pulmonary Embolism Using Inflammatory Biomarkers and the Simplified Pulmonary Embolism Severity Index
Source: J Clin Med. 2026 Jun 11;15(12):4531. doi: 10.3390/jcm15124531 (PMC13302099; doi:10.3390/jcm15124531)
Supplement: Supplementary file 1 [file jcm-15-04531-s001.zip › Table S2-Tripod-Checklist-Prediction-Model-Development-and-Validation.pdf]

TRIPOD Checklist: Prediction Model Development and Validation

Section/Topic	Item		Checklist Item	Page
**Title and abstract**				
Title	1	D;V	Identify the study as developing and/or validating a multivariable prediction model, the target population, and the outcome to be predicted.	Title: “Nomogram Based Mortality Prediction in Acute Pulmonary Embolism Using Inflammatory Biomarkers and the Simplified Pulmonary Embolism Severity Index”
Abstract	2	D;V	Provide a summary of objectives, study design, setting, participants, sample size, predictors, outcome, statistical analysis, results, and conclusions.	Abstract (page 1)
**Introduction**				
Background and objectives	3a	D;V	Explain the medical context (including whether diagnostic or prognostic) and rationale for developing or validating the multivariable prediction model, including references to existing models.	Introduction (section 1, pages 2–3)
	3b	D;V	Specify the objectives, including whether the study describes the development or validation of the model or both.	Introduction, final paragraph: “develop an integrated risk prediction model (nomogram)”
**Methods**				
Source of data	4a	D;V	Describe the study design or source of data (e.g., randomized trial, cohort, or registry data), separately for the development and validation data sets, if applicable.	Section 2.1 (multicenter retrospective cohort) – development only; external validation not performed.
	4b	D;V	Specify the key study dates, including start of accrual; end of accrual; and, if applicable, end of follow-up.	Section 2.1: “September 1, 2018, and September 1, 2024” for diagnosis; follow up until September 1, 2025.
Participants	5a	D;V	Specify key elements of the study setting (e.g., primary care, secondary care, general population) including number and location of centres.	Section 2.1: three tertiary care centres in Turkey.
	5b	D;V	Describe eligibility criteria for participants.	Section 2.2: inclusion and exclusion criteria.
	5c	D;V	Give details of treatments received, if relevant.	Table 1A (thrombolytic treatment, anticoagulation added in revision). Section 2.3 mentions medications.
Outcome	6a	D;V	Clearly define the outcome that is predicted by the prediction model, including how and when assessed.	Section 2.4: primary outcome 12 month all cause mortality, secondary 30 day and 90 day mortality; from hospital records
	6b	D;V	Report any actions to blind assessment of the outcome to be predicted.	Not explicitly blinded (retrospective, but outcome is objective mortality). Mentioned in limitations.
Predictors	7a	D;V	Clearly define all predictors used in developing or validating the multivariable prediction model, including how and when they were measured.	Section 2.5 (blood sampling within 4 hours), Section 2.6 (calculation of indices), Section 2.7 (sPESI).
	7b	D;V	Report any actions to blind assessment of predictors for the outcome and other predictors.	Retrospective, predictors from routine labs; no blinding mentioned. Not required.
Sample size	8	D;V	Explain how the study size was arrived at.	No formal calculation; all eligible patients included. Stated in Section 2.2 and Figure 1.
Missing data	9	D;V	Describe how missing data were handled (e.g., complete-case analysis, single imputation, multiple imputation) with details of any imputation method.	Complete case analysis; patients with missing data excluded (n=179). Section 2.2 and 2.3.
Statistical analysis methods	10a	D	Describe how predictors were handled in the analyses.	Section 2.8: ROC analysis, Cox regression, logistic regression; continuous variables used as continuous or dichotomised using Youden cut offs.
	10b	D	Specify type of model, all model-building procedures (including any predictor selection), and method for internal validation.	Section 2.9: multivariable Cox and binomial logistic regression; composite risk score; internal validation with
	10c	V	For validation, describe how the predictions were calculated.	Not applicable (external validation not done).
	10d	D;V	Specify all measures used to assess model performance and,	Section 3.2 (AUC, sensitivity, specificity), Section 3.7 (C index, optimism corrected C index, calibration, Hosmer

			if relevant, to compare multiple models.	Lemeshow test, accuracy, PPV, NPV, NRI/IDI in supplementary).
	10e	V	Describe any model updating (e.g., recalibration) arising from the validation, if done.	Not applicable.
Risk groups	11	D;V	Provide details on how risk groups were created, if done.	Section 3.6: composite inflammatory risk score (0–10) and sPESI categories; nomogram points.
Development vs. validation	12	V	For validation, identify any differences from the development data in setting, eligibility criteria, outcome, and predictors.	Not applicable (no external validation).
**Results**				
Participants	13a	D;V	Describe the flow of participants through the study, including the number of participants with and without the outcome and, if applicable, a summary of the follow-up time. A diagram may be helpful.	Figure 1 (flowchart), Section 3.1 (338 patients, 149 deaths), Section 3.4 (person time analysis).
	13b	D;V	Describe the characteristics of the participants (basic demographics, clinical features, available predictors), including the number of participants with missing data for predictors and outcome.	Table 1A (demographics, comorbidities, sPESI), Table 1B (inflammatory indices); missing data described in Section 2.2 and Figure 1.
	13c	V	For validation, show a comparison with the development data of the distribution of important variables (demographics, predictors and outcome).	Not applicable.
Model development	14a	D	Specify the number of participants and outcome events in each analysis.	Section 3.1 (N=338, events=149), Section 3.5 (30 day and 90 day events).
	14b	D	If done, report the unadjusted association between each candidate predictor and outcome.	Table 1A and 1B (comparison survivors vs. non survivors), univariable Cox in Table 5.
Model specification	15a	D	Present the full prediction model to allow predictions for individuals (i.e., all regression coefficients, and model intercept or baseline survival at a given time point).	Table 6 (logistic regression coefficients and OR), Figure 5 (nomogram points).
	15b	D	Explain how to the use the prediction model.	Section 3.6 and Figure 5 (explanation of points and risk calculation).
Model performance	16	D;V	Report performance measures (with CIs) for the prediction model.	Section 3.2 (AUC for inflammatory indices), Section 3.7 (AUC 0.806, 95% CI 0.761–0.851; optimism corrected
Model-updating	17	V	If done, report the results from any model updating (i.e., model specification, model performance).	Not applicable.
**Discussion**				
Limitations	18	D;V	Discuss any limitations of the study (such as nonrepresentative sample, few events per predictor, missing data).	Limitations section (after Discussion).
Interpretation	19a	V	For validation, discuss the results with reference to performance in the development data, and any other validation data.	Not applicable (no external validation).
	19b	D;V	Give an overall interpretation of the results, considering objectives, limitations, results from similar studies, and other relevant evidence.	Discussion throughout, especially final paragraphs.
Implications	20	D;V	Discuss the potential clinical use of the model and implications for future research.	Discussion (e.g., "This approach offers a practical and low cost solution…"), Conclusion.
**Other information**				
Supplementary information	21	D;V	Provide information about the availability of supplementary resources, such as study protocol, Web calculator, and data sets.	Supplementary material (TRIPOD checklist, additional figures/tables). Data available from corresponding author.

Funding	22	D;V	Give the source of funding and the role of the funders for the present study.	Funding" statement (no external funding).

*Items relevant only to the development of a prediction model are denoted by D, items relating solely to a validation of a prediction model are denoted by V, and items relating to both are denoted D;V. We recommend using the TRIPOD Checklist in conjunction with the TRIPOD Explanation and Elaboration document.
